# Low dose effect of bisphosphonates on hMSCs osteogenic response to titanium surface *in vitro*

**DOI:** 10.1016/j.bonr.2017.02.002

**Published:** 2017-02-16

**Authors:** N.R. Alqhtani, N.J. Logan, S. Meghji, R. Leeson, P.M. Brett

**Affiliations:** a University College London, Eastman Dental Institute, 256 Gray's Inn Road, London WC1X 8LD, UK; b Department of Oral and Maxillofacial Surgery and Diagnostic Sciences, College of Dentistry, Sattam bin Abdulaziz University, AlKharj, Saudi Arabia

**Keywords:** Bone remodelling/regeneration, Human mesenchymal stem cells, Bisphosphonates, Titanium

## Abstract

Since the 1980s, titanium (Ti) implants have been routinely used to replace missing teeth. This success is mainly due to the good biocompatibility of Ti and the phenomenon of osseointegration, with very early events at implant placement being important in determining good osseointegration. However, enhancing implant performance with coatings such as hydroxyapatite (HA) and calcium phosphate has proved largely unsuccessful. Human mesenchymal stem cells (hMSCs) are the first osteogenic cells to colonise implant surfaces and offer a target for enhancing osseointegration. We previously reported that small doses of bisphosphonate (BP) may play an integral role in enhancing hMSC proliferation and osteogenic differentiation. The aim of this study is to investigate whether small doses of bisphosphonates enhance proliferation and osteogenic differentiation of hMSCs on Ti surfaces, to enhance bone osseointegration and to accelerate wound healing around the implant surface. Our data suggests that treating cells with small doses of BP (100 nM & 10 nM) induces significant hMSC stimulation of osteogenic markers including calcium, collagen type I and ALP compared to control group on titanium surfaces (P < 0.05). In addition, cell proliferation and migration were significantly enhanced on titanium surfaces (P < 0.05).

## Highlights

• Bisphosphonates appear to have a significant impact on hMSCs migration towards Ti surfaces.
• Bisphosphonates exerted effects on hMSC morphology; cells had increase spreading and adhered to Ti surfaces.
• Bisphosphonates exert stimulatory effects on hMSC osteogenic differentiation.

## 1 Introduction

In recent decades, titanium (Ti) implants have been successfully used to replace missing teeth. This success is mainly due to the good biocompatibility of Ti and the phenomenon of osseointegration. Following implantation, the surrounding tissue may respond in two ways; formation of fibrous tissue or formation of new bone. Formation of fibrous tissue around the implant will lead to the clinical failure of the implant, whereas formation of new bone on the implant surface without interfering connective tissue is the desired outcome (Baas et al., 2012). The surface properties of the implant play an integral role for implant osseointegration (Le et al., 2007, Colombo et al., 2012). Also, the topography and chemistry of the surface properties can control the quantity and quality of adhered cells to the implant (Rosales-Leal et al., 2010, Logan and Brett, 2013). It has been reported that optimal surface roughness can lead to effective osseointegration (Halldin et al., 2014). This effect was due to increased osteoblast proliferation, differentiation and matrix protein production, *e.g.* collagen type I. Furthermore, surface hydrophilicity enhances cell interaction and adhesion to dental implants (Zhao et al., 2005, Logan et al., 2014a).

The coating of metal dental implants with different bone inducing materials can induce positive effects. These materials include, bone stimulating factors, bisphosphonates, fluoride, HA and calcium phosphate, and titanium/titanium oxide (Logan et al., 2014b, Xuereb et al., 2015). Calcium phosphate (CaP) which is mainly composed of hydroxyapatite has been shown to induce osteogenic differentiation and osteoblasts growth on implant surfaces (Yang, 2001). It has been reported that coating implants with growth factors such as bone morphogenetic protein (BMP) stimulates bone formation and improves implant success (Wikesjo et al., 2008). HA also accelerates new bone formation by activation of osteoblast proliferation and differentiation (Kweon et al., 2014, Lee et al., 2014, Yang et al., 2010). However, the long-term survival of HA-coated dental implants is still a controversial clinical issue. Plasma spraying is a commonly used technique to coat implants, but this technique has the disadvantage of requiring substantially thick coating layers and furthermore, controlling the final coating composition is difficult. However, titanium implants are widely used in the clinic because of their strength, low stiffness and light weight.

Bisphosphonates are chemical analogues related to pyrophosphate; they are well-known inhibitors of osteoclast activity. Over the last 40 years, they been used in the clinic to treat various bone diseases characterised by excessive bone resorption such as osteoporosis, malignant bone disease and hypercalcaemia of malignancy (Dominguez et al., 2011, Russell, 2011, Russell et al., 2007). Osteoporosis is one of the most common skeletal diseases affecting the quality of all bones, including the jaw(Berruti et al., 2001). Human mesenchymal stem cells (hMSCs) show regenerative properties and are able to differentiate into different cell types such as osteoblasts (Kobolak et al., 2015). We have shown that various types of bisphosphonates promote osteogenic differentiation of hMSCs *in vitro* (Alqhtani et al., 2014). These findings suggest that bisphosphonates may have a direct stimulus on osteoblast mineralisation. It has also been reported that coating implants with bisphosphonate-eluting fibrinogen may improve implant osseointegration, but there are risks in developing chronic osteomyelitis (Abtahi et al., 2012).

To our knowledge, the systemic application of low doses (less than 1000th of clinical doses) of bisphosphonate has not been tested on hMSC proliferation and osteogenic differentiation on titanium implants. With this in mind, the aim of this study was to investigate whether low doses of bisphosphonate enhanced proliferation and osteogenic differentiation of hMSCs on Ti surfaces. Furthermore, we investigated whether application of these bisphosphonates (pamidronate and alendronate) enhanced bone quality, improved implant osseointegration and accelerated wound healing around the surface of the implant. We investigated the effect of these drugs on cell proliferation, migration, adhesion and attachment. Osteogenic differentiation markers were measured including calcium, collagen type I and alkaline phosphates activity (ALP).

## 2 Materials and methods

### 2.1 Titanium discs preparation

Two commonly used bisphosphonates were assessed; alendronate (ALE) and pamidronate (PAM) (Sigma-Aldrich) on hMSC proliferation and osteogenic response to Ti surfaces. 15 mm diameter polished titanium discs were used and were designed to fit into the wells of 24 well tissue culture plates (Sarstedt). Surface roughness was analysed using laser profilometry (Scranton, Proscan 1000). Contact angle measurements were performed using an optical contact angle meter (KSV Instruments Ltd., CAM200).

### 2.2 Cell culture

hMSCs were cultured in 150 cm^2^ tissue culture flasks (Sarstedt) in minimum essential medium (α-MEM, Gibco BRL) supplemented with 10% foetal bovine serum (FBS) (Invitrogen) and 100 U/ml of penicillin/streptomycin (P/S) (Sigma-Aldrich). hMSCs were incubated at 37 °C at 5% CO_2_ and harvested for experiments once they reached 80% confluence. To promote hMSCs osteogenic differentiation, osteogenic media (OM) comprising of Dulbecco's modified Eagle's medium (DMEM) low glucose pyruvate (Gibco), 10% foetal bovine serum, 100 U/ml P/S, and further supplemented with β-glycerol phosphate (Sigma-Aldrich), ascorbic acid (Sigma-Aldrich), and dexamethasone (Sigma-Aldrich) was used.

### 2.3 Proliferation

hMSCs were seeded at a density of 1 × 10^4^ cells/well in 1.9-cm^2^ wells containing growth media and incubated at 37 °C at 5% CO_2_. The next day, the media was removed and changed to growth (GM) media and media supplemented with different concentrations of alendronate and pamidronate (100 nM & 10 nM). Proliferation of cells was assessed using the Alamar blue assay (AbD Serotec). A 10% dye solution was added to each well and cells incubated for 4 h at 37 °C at 5% CO_2_. 100 μl aliquots were transferred into 96 well black plates (Fisher Scientific) to measure fluorescence intensity (Excitation λ = 530 nm, emission λ = 590 nm) using a plate reader (BioTeK FLX800). Interpolation with a standard plate was used to generate the cell numbers.

### 2.4 Cell migration

Cell migration assays (Cultrex, 3465-.24-k) were used according to the manufacturer's instructions to assess the migration of cells towards Ti surfaces in the presence of BPs. Briefly, hMSCs were cultured in serum free media 24 h prior to the assay. The next day, 10 × 10^4^ cells per well (n = 3) were seeded onto an 8 μm pore polymer membrane in an insert chamber to facilitate cell migration. A 500 μl aliquot of GM was placed at the bottom of the chamber and cells incubated at 37 °C at 5% CO_2_ for 4.5 h. Membranes were washed three times with wash buffer and incubated with cell dissociation solution containing calcien AM at 37 °C for 60 min. Then the fluorescence intensity (Excitation λ = 530 nm, emission λ = 590 nm) was measured on a plate reader (BioTeK FLX800). Interpolation with a standard plate was used to generate the cell numbers.

### 2.5 Calcium deposition

The quantichrom calcium kit (BioAssay Systems) was used according to manufacture instructions. Briefly, cell monolayers were washed with PBS then lysed with 500 μl 1 M HCl for ≤ 1 h after which 5 μl of lysate was transferred to a clear bottomed 96-well plate. To this, 200 μl of freshly prepared working reagent was added. Absorbances were read at 612 nm using a plate reader (Tecan, M200).

### 2.6 Extracellular collagen deposition

Collagen deposition was assayed using the Sirocel kit according to manufacturer's instructions (Biocolor). Briefly, cell monolayers were lysed with cold 0.5 M acetic acid supplemented with 100 μg/ml porcine pepsin. Samples were incubated overnight with 200 μl isolation and concentration reagent at 4 °C. Concentrated samples were centrifuged at 12,000 revolutions per minute (rpm) for 10 min to pellet collagen. Pellets were stained with Sirius Red stain for 30 min, and then centrifuged at 12,000 rpm for 10 min. The pellets were washed in acid-salt solution to remove excess dye. The stain was eluted in 250 μl alkali solution. Absorbances were measured at 555 nm using a plate reader (Tecan, M200).

### 2.7 Alkaline phosphatase activity

ALP activity was assessed using the colorimetric SensoLyte® pNPP ALP Assay Kit (Cambridge Bioscience) according to the manufacturer's instructions. Briefly, 1 × 10^4^ cells/well were seeded into 24-well plates and grown for 24 h at 37 °C at 5% CO_2._ The media was changed to OM supplemented with different concentrations (100 nM & 10 nM) of the drug (ALE and PAM). At day 7, the cells were washed twice in PBS, lysed with 500 μl 0.1% triton X-100, scraped into microcentrifuge tubes and incubated at 4 °C for 10 min. The cell suspension was centrifuged at 2500 ×* g* for 10 min at 4 °C. For enzyme activity, the standards and cell lysates were incubated at 37 °C for 1 h, and then transferred to a 96-well plate and absorbances read at 405 nm using a plate reader (Tecan, M200).

### 2.8 Immunocytochemistry and cell morphology

To analyse the effects of low BP doses (100 nM & 10 nM) on cell adhesion and attachment, focal adhesion and cytoskeletal proteins, vinculin and F-actin were studied using fluorescence microscopy. Cells were grown in 24 well plates and incubated with ALE and PAM in OM for 24 h. The next day, the medium was discarded, the cells washed with PBS and fixed with 4% paraformaldehyde for 30 min. Vinculin and f-actin were permeabilise using triton × 100. The cells were rinsed with PBS, blocked with 10% goat serum in PBS for 30 min at room temperature, and incubated with anti-vinculin antibody (Abcam) at 1:200 dilution and left at 4 °C overnight. Next day, after several PBS washes, the cells were incubated with goat anti-mouse IgG 568 (Life Technologies) at 1:200 dilution for 1 h at room temperature in the dark. The cells were washed three times in PBS (5 min each) and stained with 4′,6-diamidino-2-phenylindole (DAPI) for nuclear visualisation. For F-actin, MSCs were stained with phalloidin-iFlour 488 in PBS (Abcam, 1:1000). Finally, the cells were washed and viewed using a Leica – DMIRB fluorescence microscope equipped with COOLSNAP Monochrome Camera. Images were collected and processed with ImageJ software and quantifiable analysis performed using the same software.

### 2.9 Scanning electron microscopy

Scanning electron microscopy (SEM) was used to analyse cell morphology. Cells were washed three times in cold PBS and fixed in 3% glutaraldehyde in 0.1 M cacodylate buffer for 24 h at 4 °C. The next day, fixative was removed and the cells dehydrated in a series of graded ethyl alcohols for 10 min at each concentration, and finally dried with hexamethyldisilazane for 2 to 5 min with subsequent removal and drying for at least an hour. The dried samples were mounted on stubs, coated with gold, and viewed using a FEI XL30 FEGSEM (FEI UK, UK).

### 2.10 Statistical analysis

hMSCs from three donors (N = 3) were used in triplicate (n = 3). Surface roughness analysis were performed at n = 5, contact angle measurement was performed at n = 9. Statistical analysis was carried out using one-way analysis of variance (ANOVA) followed by a Bonferroni post-test, with P < 0.05 considered to be statistically significant.

## 3 Results

### 3.1 Surface properties

As shown in Table 1, surface roughness were analysed where the mean of R_a_ value = 0.63 μm (n = 5) and the contact angle were performed with a mean of 69.1 (n = 9).

**Table 1:** Surface roughness and contact angle of Ti surface.

	Mean	± Standard deviation
Surface roughness	0.63 μm	± 0.004 μm
Contact angle	69.1°	± 3.7°

### 3.2 Proliferation

**Fig. 1:**
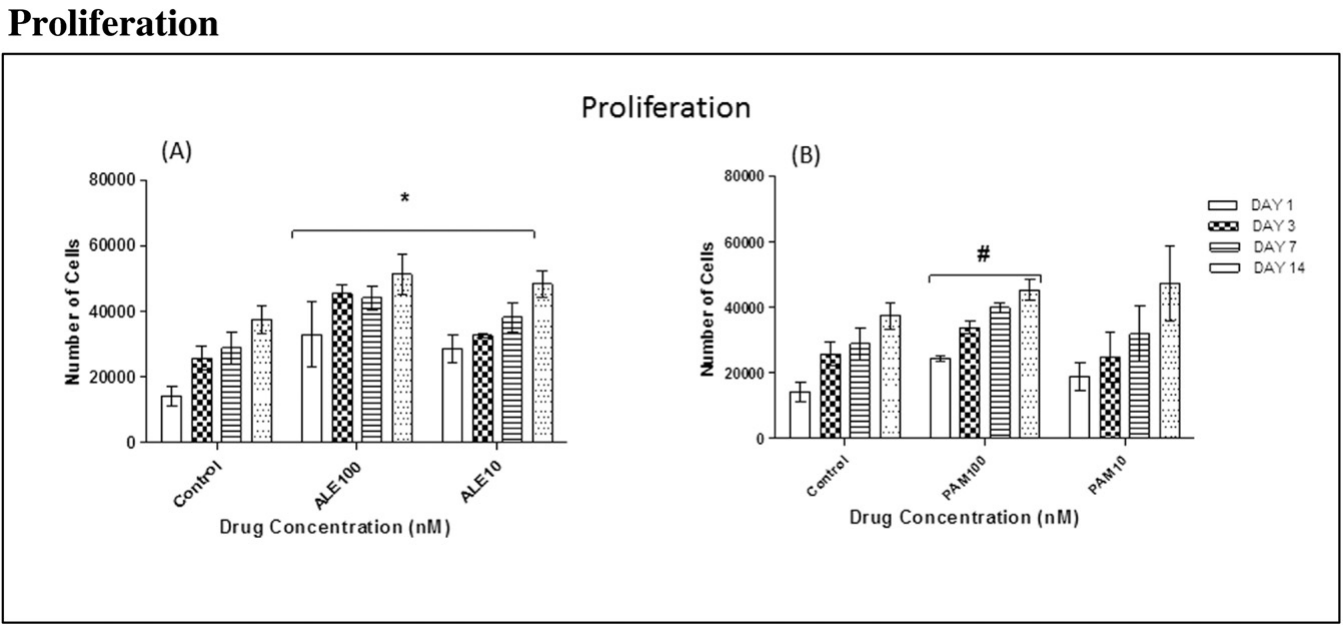
The effects of low doses of ALE and PAM (100 nM and 10 nM) on hMSC proliferation on Ti surfaces were assessed at different time points. (A): All groups treated with ALE (100 nM and 10 nM) had shown significant cell proliferation when compared to the control group that was treated with GM only on day 1, 3, 7 and 14. (B): The group treated with 100 nM showed significant cell proliferation compared to the control group that treated with GM only on day 1, 3, 7 and 14 (values = mean ± SD; P < 0.05).

Human MSC proliferation in GM was examined over 1, 3, 7 and 14 days. ALE (100 nM and 10 nM) stimulated significant cell proliferation in all cells on days 1, 3, 7 and 14 (Fig. 1(A)). Also, cells that had been treated with 100 nM PAM showed significant proliferation when compared to control cells which were treated with GM only. Cells treated with 10 nM PAM promoted cell proliferation but it was not statistically significant (Fig. 1(B)).

### 3.3 Migration

**Fig. 2:**
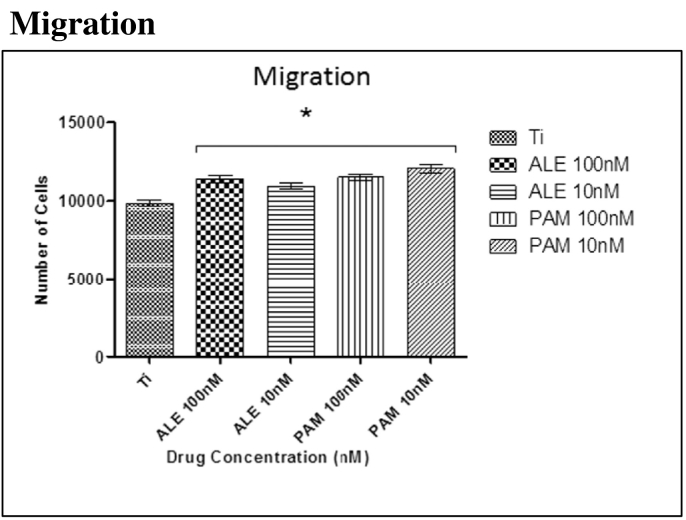
Significant hMSC migration was observed after 4.5 h incubation at 37 °C at 5% CO_2_. Significant differences were observed between the cells treated with the low dose of drugs and control group (OM only). Each column represents the mean ± SD.

Cells after 4.5 h at 37 °C at 5% CO_2_, were observed to have migrated towards the substrate. However, cells with low doses of BPs (100 nM and 10 nM) have shown that these drugs significantly enhanced cell migration towards titanium surface when compared to the control group that had been treated with OM only (Fig. 2).

### 3.4 Mineralisation

**Fig. 3:**
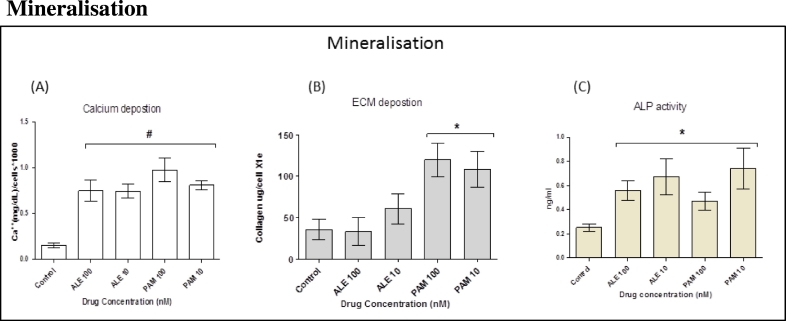
Cells were divided into five groups with each group treated with different ALE and PAM concentration (100 nM and 10 nM). (A): At day 21, calcium deposition was analysed. The results showed that BPs significantly stimulated hMSC mineralisation following drug treatment for 21 days when compared to cells that had been treated with OM only. (B): At day 14, extracellular collagen formation was analysed. The results showed that PAM significantly stimulated hMSC to produce collagen following treatment with drugs for 14 days when compared to cells that had been treated with OM only. No significant changes were observed in cells treated with ALE when compared to the control. (C): ALP activity after 7 days incubation was significantly increased in all cells treated with the lower dose of BPs drug when compared to the control group treated with OM only. Each column represents the mean ± SD.

Markers that were linked to early and late stage osteogenesis were analysed. These markers included calcium, collagen type I and ALP. Data showed that both BP drugs significantly stimulated hMSC osteogenesis following drug treatment for 3 weeks when compared to cells that had been treated with OM only (Fig. 3(A) and (B)). However, there was no significant difference in collagen deposition for cells that had been treated with ALE (Fig. 3(B)). Furthermore, ALP is a useful early marker in osteogenesis and is believed to be part of the mineralisation process. Data showed that treating hMSCs with ALE and PAM for 7 days caused significant increases in ALP activity (Fig. 3(C)).

### 3.5 Immunocytochemistry and cell morphology

**Fig. 4:**
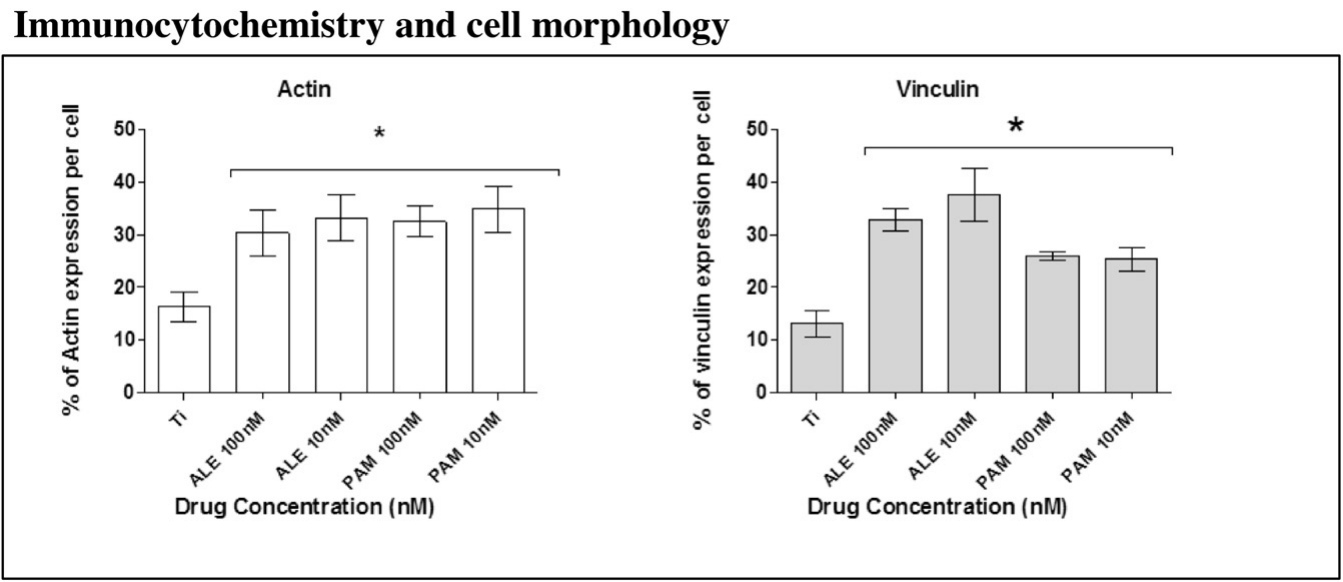
Actin and vinculin expression analysis after 24 h of culture in OM with and without BPs. Groups that been treated with low dose of ALE and PAM showed a significant increase in actin and vinculin expression compared to the control group that was treated with only OM. Each column represents the mean ± SD.

**Fig. 5:**
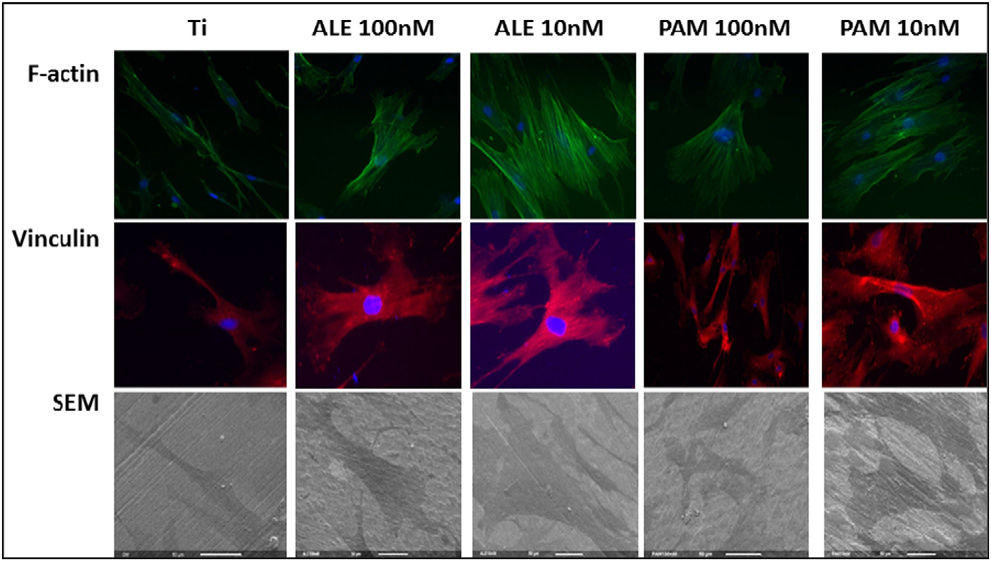
Fluorescence microscopy images showing the effect of low dose of drugs on cell organisation and spreading on Ti surfaces after 24 h. Actin expression (green images) appeared more abundant in cells treated with the ALE and PAM (100 nM and 10 nM). Likewise, vinculin expression (red images) appeared more abundant in treated cells. Images were taken using a × 40 objective. Scale bar = 50 μM. SEM images were found to correlate with fluorescent images.

Cell morphology was analysed by labelling cells with phalloidin to visualise internal F-actin structures. Data showed that after 24 h of culture, those cells treated with ALN and PAM, exhibited clear differences in cell appearance and spread. These finding were supported by SEM images (Fig. 4, Fig. 5).

## 4 Discussion

Bone remodelling is a complex process requiring several cellular processes; bone cell recruitment, differentiation, bone synthesis and angiogenesis to stimulate blood flow (Seeman, 2009). Good biocompatibility and rapid osseointegration are essential elements of implant success and stability (Goriainov et al., 2014, Plecko et al., 2012, Quirynen et al., 2014, Von et al., 2014). In this study, we investigated whether low doses of BPs enhanced proliferation and osteogenic differentiation of hMSCs on Ti surfaces. hMSCs were used as they are the first cells to colonise implant surfaces and offer a target for enhancing osseointegration (Davies, 2003).

Topographical and chemical modifications of implant materials play integral roles in cellular migration, attachment, adhesion and proliferation of hMSCs (Logan et al., 2015, Wall et al., 2009, Wang et al., 2008). The ability of BPs to promote hMSCs proliferation on Ti surfaces is a crucial factor in implant success. In this study, we observed that over time, low doses of BP increased cell density at Ti surfaces. This observation may influence bone formation and improve osseointegration. In addition, these findings suggest that ALE and PAM exert effects on stem cell density. Furthermore, BPs appear to have a significant impact on hMSCs migration towards Ti surfaces. This effect may not be solely due to cell proliferation, as our data indicates that BPs have an effect at the cellular level by accelerating cell migration towards Ti surfaces. These results are supported by enhanced adhesion and cell spreading (Fig. 4, Fig. 5).

SEM images showed that the ALE and PAM exerted effects on hMSC morphology; cells were spread and adhered to Ti surfaces. This was confirmed by the expression of focal adhesion proteins; vinculin spreading is considered a parameter of the interaction between the cell and implant material (Ezzell et al., 1997, Logan and Brett, 2013). A firm attachment and spread of hMSCs is an important factor for differentiation to osteoblasts, which over time become mature and produce fibronectin (Terheyden et al., 2012). From these findings, we believe that the ALE and PAM may have stimulatory effect on chemotactic behaviour towards substrates such as Ti.

To evaluate BPs on hMSC mineralisation, both early and late osteogenic markers such as calcium, collagen type I and ALP activity were assessed. It has been shown that in a clinical setting, following implantation, several cell types migrate towards the implant surface including endothelial cells, osteoblasts and stem cells (Khalil et al., 2011). This is a very important step for osteoinduction and over time, this accumulation at the implant surface will enhance implant stability and its success (Javed et al., 2013, Truhlar et al., 2000).

Our data have shown that BPs exert stimulatory effects on hMSC osteogenic differentiation; cells treated with drugs produce more calcium when compared to the control group. These results were supported by elevation of ALP activity for both drugs after 7 days in culture. However, collagen type I is the main component of the extracellular matrix and is produced by osteoblasts and other cells derived from hMSCs (Gelse et al., 2003). In this work, PAM stimulated higher collagen type I formation on Ti surface compared to ALE; this could be that PAM is clinically more potent than ALE. The clinical success of any implant is based on its ability to anchor the endosseous implant to the surrounding bone (Chrcanovic et al., 2015).

This research confirms that low concentrations of ALE and PAM enhance the hMSC osteogenic response *in vitro*, and therefore could have translational applications *in vivo*; enhancing osseointegration and clinical outcomes for Ti and other bone implants. We found that systemic application of low drug doses, which is critically, almost 1000 times less than clinical doses and coating concentrations, has a largely beneficial effect on hMSC proliferation and osteogenic differentiation on Ti surfaces. Previous reports have shown that coating with BPs inhibits the osteolysis process (Abtahi et al., 2012, Stadelmann et al., 2008). In contrast to these reports, in our study, we have assessed hMSC migration, proliferation and osteogenic differentiation and used recognised assays and biomarkers to explore this phenomenon.

Apart from BP, different materials have also been used to stimulate bone regeneration and formation such as guided bone regeneration (GBR) (Donos et al., 2004, Elgali et al., 2015). Systemic application of this drug in conjunction with regenerative materials such as the GBR may synergistically enhance the bone remodelling process and accelerate wound healing. These findings may enhance bone remodelling and wound healing in the peri-implant area.

In conclusion, we have demonstrated that low doses of BPs stimulate hMSCs proliferation and osteogenic differentiation on Ti surfaces. These findings have promising implications in the systemic application of low doses of these BP drugs towards improved implant osseointegration and successful clinical outcomes. We believe that the systemic application of BP drugs could be pivotal in accelerating osseointegration and the bone healing process.

## Conflict of interest

The authors of this publication confirm that there are no conflicts of interest.
